# κ- and λ-Carrageenans from Marine Alga *Chondrus armatus* Exhibit Anticancer In Vitro Activity in Human Gastrointestinal Cancers Models

**DOI:** 10.3390/md20120741

**Published:** 2022-11-25

**Authors:** Vladlena A. Tiasto, Nikolay V. Goncharov, Alexander O. Romanishin, Maxim E. Zhidkov, Yuri S. Khotimchenko

**Affiliations:** 1 Institute of Life Sciences and Biomedicine, Far Eastern Federal University, 690922 Vladivostok, Russia; 2 A.V. Zhirmunsky National Scientific Center of Marine Biology, Far Eastern Branch, Russian Academy of Sciences, 690041 Vladivostok, Russia; 3 School of Life Sciences, Immanuel Kant Baltic Federal University, 236041 Kaliningrad, Russia; 4 Institute of High Technologies and Advanced Materials, Far Eastern Federal University, 690922 Vladivostok, Russia

**Keywords:** antitumor activity, oligosaccharides, carrageenans, *Chondrus armatus*, colon cancer, esophageal carcinoma, cell cycle

## Abstract

The carrageenans isolated from red algae demonstrated a variety of activities from antiviral and immunomodulatory to antitumor. The diverse structure and sulfation profile of carrageenans provide a great landscape for drug development. In this study, we isolated, purified and structurally characterized κo- and λo- oligosaccharides from the marine algae *Chondrus armatus*. We further examined the tumor suppressive activity of both carrageenans in gastrointestinal cancer models. Thus, using MTT assay, we could demonstrate a pronounced antiproliferative effect of the carrageenans in KYSE-30 and FLO-1 as well as HCT-116 and RKO cell lines with IC_50_ 184~405 μg/mL, while both compounds were less active in non-cancer epithelial cells RPE-1. This effect was stipulated by the inhibition of cell cycle progression in the cancer cells. Specifically, flow cytometry revealed an S phase delay in FLO-1 and HCT-116 cells under κo-carrageenan treatment, while KYSE-30 demonstrated a pronounced G_2_/M cell cycle delay. In line with this, western blotting revealed a reduction of cell cycle markers CDK2 and E2F2. Interestingly, κo-carrageenan inhibited cell cycle progression of RKO cells in G_1_ phase. Finally, isolated κo- and λo- carrageenans induced apoptosis on adenocarcinomas, specifically with high apoptosis induction in RKO cells. Overall, our data underline the potential of κo- and λo- carrageenans for colon and esophageal carcinoma drug development.

## 1. Introduction

The route to drug discovery based on the utilization of marine natural products still continues to be attractive. Today, a wide range of available commercial drugs are of natural origin [1]. The ocean is a source of accessible and unique resources for the exploration of beneficial features for biomedical applications [2]. In particular, seaweed contains many valuable components with great anti-cancer potential [3,4,5]. Carrageenans isolated from red seaweed have been the object of drug discovery for the past few decades [6]. The chemical structure of carrageenans is based on disaccharide repeating units, consisting of D-galactose residues connected by regularly alternating β-(1-4) and α-(1-3) glycosidic bonds [7]. The two main types of carrageenan are κ- and λ-, which are different in sulfation profile: κ-carrageenan contains one sulfate group per two molecules of galactose, while λ- carrageenan contains three sulfate groups per two molecules of galactose. In these polymers, the content of sulfate groups varies from 20% in κ-carrageenan to 41% in λ-carrageenan [8]. This variety of sulfate content determines a wide range of biological activity [9,10,11].

While the most studied effects of marine polysaccharides are antiviral, immunomodulatory, and antioxidant [12,13,14], some studies observed the ability of carrageenans to suppress the growth of different types of malignant tumors. It was found that κ- and λ- carrageenans from red seaweed *Chondrus ocellatus* inhibited the proliferation of H-22 cell line [15,16], from *Kappaphycus striatum* [17] inhibited proliferation of BGC and Hela cell lines. Carrageenans from *Hypnea musciformis* inhibited LM2 and SH-SY5Y cell lines [18,19], and those from *Kappaphycus alvarezii* had the same effect on Caco-2 and HepG2 [20]. The wide range of biological activities of *Chondrus armatus* carrageenans have been studied, including immunomodulatory [21] and anti-inflammatory properties [22]; moreover, there is strong evidence determining the antitumor potential of *Chondrus armatus* carrageenans [23,24]. However, the detailed mechanisms of carrageenan suppressive activity on tumor cells remain unexplained. Despite many examples of the tumor suppressive activities of carrageenans from different seaweeds, development of drugs based on this data remains impossible. Moreover, none of the studies have systematically observed the antitumor potential of carrageenans for specific types of cancer. In our view, the antitumor properties of carrageenans must be first studied on cancer models that reproduce similar features to specific human tumor types. 

In this study, we observed the antitumor potential of κo- and λo- carrageenans (oligosaccharides of κ- and λ- carrageenans) for gastrointestinal cancers that continue to be a leading cause of death worldwide [25]. Here we isolated and structurally characterized κo- and λo-carrageenans from *Chondrus armatus*. We identified the changes in cell cycle progression upon treatment with carrageenans on human colon cancer cell lines and esophageal carcinoma cell lines.

## 2. Results

### 2.1. Isolation, Purification, and Structural Characterization of κ- and λ-Carrageenans from Chondrus armatus

**Figure 1:**
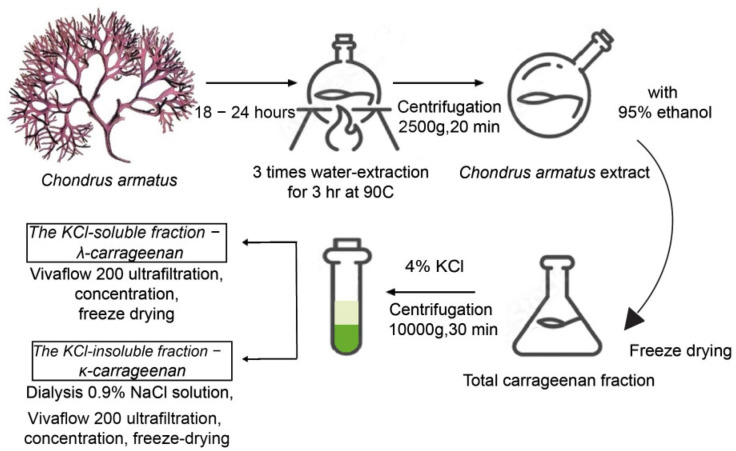
Isolation and purification of carrageenans and their structural features. The dried algae were soaked in 96% alcohol and then underwent water extraction three times. The obtained extracts were combined, filtered, and centrifuged at 2500× *g*, 10 °C for 20 min. Polysaccharides were precipitated with three times greated volume of 95% ethanol. The resulting substance was a fraction of the total carrageenan. A 4% KCl solution is added to the polysaccharides, followed by centrifugation (10,000× *g*, 30 min, 4 °C). The obtained supernatant contains soluble KCl fraction − λ carrageenan (λ-polysaccharide). The fraction insoluble in KCl represented κ-carrageenan (κ-polysaccharide). We performed sonication for depolymerization of initial κ- and λ-carrageenan polysaccharides. The resulting samples of carrageenan oligosaccharides (κo- and λo-) were dialyzed and freeze-dried.

To achieve maximum quality of the output samples we have developed a unique protocol for fractionation and isolation of the κ- and λ-carrageenans from red seaweed *C. armatus* (Figure 1).

**Figure 2:**
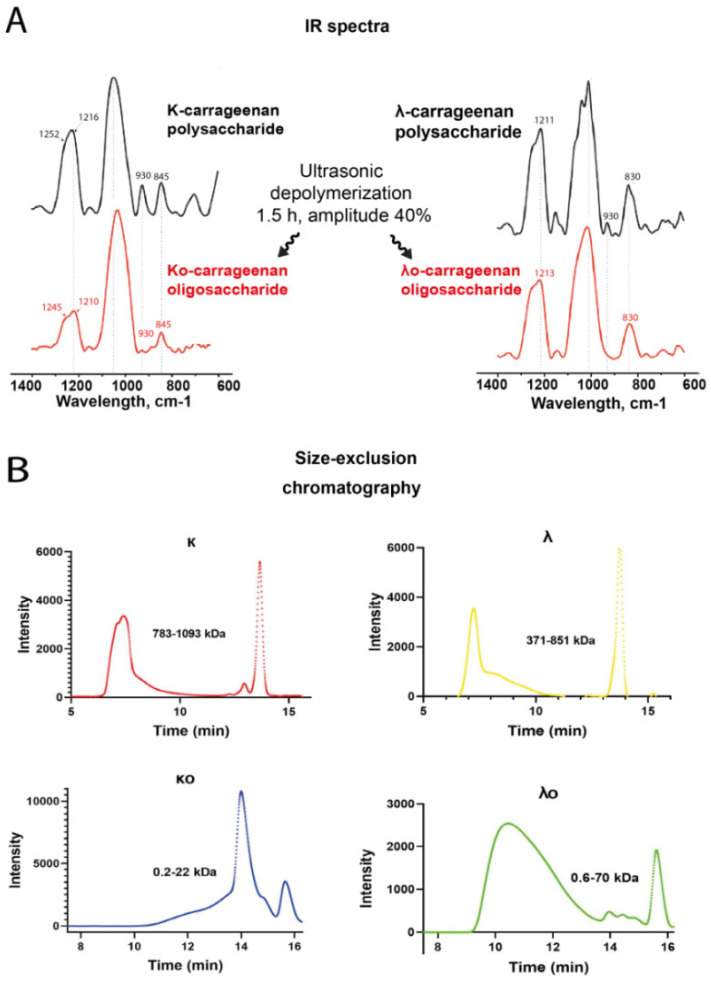
Structural features of κ- and λ-carrageenan polysaccharides and oligosaccharides (**A**) Infrared spectra. (**B**) Size exclusion chromatography and molecular weights.

The structure of the isolated oligocarrageenans was confirmed by IR spectroscopy following the polysaccharides whose structure was established earlier [26]. Using IR spectroscopy, we compared changes in κ- and λ-polysaccharides that can occur as a result of depolymerization. The resulting spectra of low molecular weight κo- and λo- carrageenans were compared with the spectra of the original κ- and λ-polysaccharides (Figure 2a). 

IR spectra demonstrated that microwave depolymerization of λ-carrageenan corresponds to the chemical structure of the initial polysaccharide. However, depolymerization of κ-carrageenan led to slight changes in the ratio of main monosaccharides (galactose and 3,6-anhydrogalactose) and a decrease in sulfate content. Moreover, the IR spectra of the isolated polysaccharides had characteristic absorption bands for κ- and λ-carrageenans. Thus, the IR spectra of κ-carrageenan and κo-carrageenan contained an absorption band at 930 cm^−1^, which corresponds to 3,6-anhydrogalactose, and a band at 845 cm^−1^, which characterizes the sulfate groups at C-4 β-D-galactose (Figure 2a). In the IR spectra of λ-carrageenan and λo-carrageenan, bands were found at 830 cm^−1^, which correspond to the sulfate group at C-6 of 4-α-galactose (Figure 2a). All spectra contained a broad absorption band at 1210–1252 cm^−1^ related to the total amount of sulfate groups [27].

NMR analysis of isolated oligosaccharides confirms IR spectra results. Chemical shifts of ^13^C NMR spectrum signals of isolated κo-carrageenan were compared to the known signals of κ-polysaccharide [28]. Two signals with intensities at 95.8 and 103.1 ppm were observed in the ^13^C NMR spectrum of the polysaccharide, which indicates the presence of two types of disaccharide units. The signal at 95.8 is characteristic of the C-1 of 1,4-linked 3,6-anhydro-α-D-galactose (DA) of κ-carrageenans. The signal at 103.1 ppm is the C-1 signal of 1,3-linked β-D-galactose 4-sulfate (G4S) of κ-carrageenans. The characteristic signals for C-2–C-6 of galactose residues of κ-carrageenan types are listed in Table 1 (Figure S1). 

In the ^13^C NMR spectrum of κo-carrageenan (*C. armatus*), the following signals are observed: the signal at 92.9 ppm corresponds to the C-1 atom of the 3,6-anhydro-D-galactose residue of i-carrageenan. The signal in the ^13^C NMR region of the spectrum of low molecular weight κo-carrageenan 105.46 ppm can be attributed to the C-1 atom of the galactose residue, which has a sulfate group at C-4 (Table 1, Figure S1).

In the case of the λo-carrageenans, comparison of chemical shifts of the signals with those known for the λ-carrageenans shows the presence of main characteristic signals of 3-linked β-D-galactopyranose 2-sulfate (G2S) and 4-linked α-D-galactopyranose 2,6-disulfate (D2S,6S) that form this type of polysaccharides [1]. It is noteworthy that the spectrum contains a single signal at 91.6 ppm, corresponding to the C-1 of D2S,6S. At the same time, instead of a single signal at 103.4 ppm of the anomeric carbon of G2S, a group of four signals is observed in the range of 101.1–104.0 ppm. ^13^C NMR data suggest that, under the action of ultrasonic treatment, apparently, partial desulfation of the G2S fragment takes place, while the D2S,6S fragments are not affected (Figure S2).

These data indicate that the main repeating units are retained despite their low molecular weight and that the cleavage of the O-glycosidic linkage is the main effect. Ultrasonication cleaves the O-glycosidic bond and reduces the molecular weight of compounds. The average molecular weights of κ- and λ-carrageenan polysaccharides determined by HPLC were 783–1093 kDa and 371–851 kDa, respectively. These samples were collected and used for obtaining low molecular weight degradation products. The average molecular weights of the κo- and λo- carrageenans were 0.2–22 kDa and 0.6–70 kDa, respectively, and were extracted from these substances by partial degradation (Table 2, Figure 2b).

According to our results, the native κ-carrageenan polysaccharide had an average molecular weight (Mw) of 784 kDa with a polydispersity index (PDI) of −3.09. At the same time, κo-carrageenan formed after sonication had a molecular weight (Mw) of 3.9 kDa with a polydispersity index (PDI) of −3.96. Thus, the Mw of the original κ-carrageenan decreased by 99.5%. As for the λ-carrageenan, the native polysaccharide λ-carrageenan had an average molecular weight (Mw) of 851 kDa with a poly-dispersity index (PDI) of −4.19, and λo-carrageenan had an average molecular weight (Mw) of 69.4 kDa with a polydispersity index (PDI) −3.24 (Table 2). Under the influence of ultrasound, the molecular weight of the λ-carrageenan decreased by 91.8%. 

Overall, we can conclude successful isolation of κo- and λo- carrageenans (Figure 2).

### 2.2. Cytotoxicity of κo- and λo-Carrageenans

**Figure 3:**
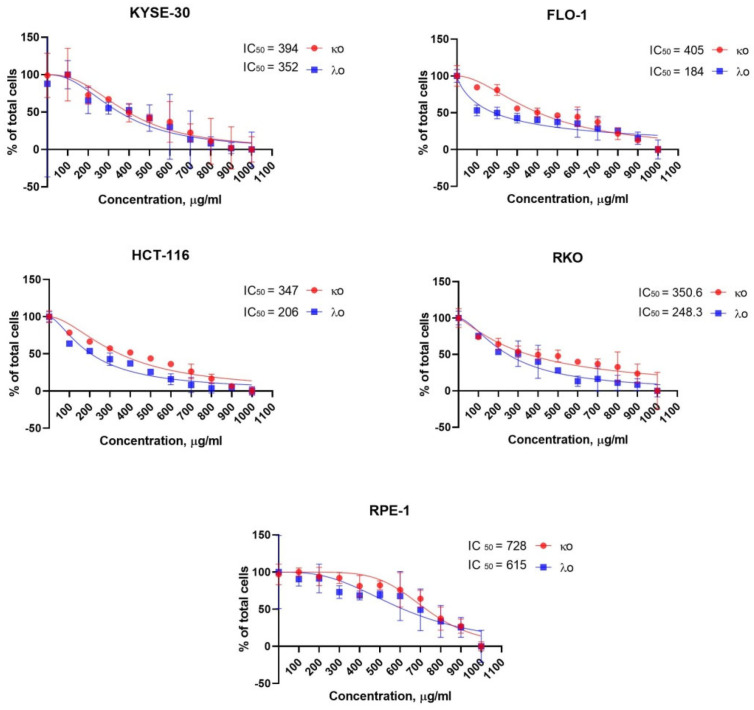
IC_50_ concentration µg/mL of carrageenans for KYSE-30, FLO-1, HCT-116, RKO, and RPE-1 cell lines.

To detect cytotoxic activities of κo- and λo-carrageenans, we used a collection of gastrointestinal cancer cell lines that reproduce similar features to specific gastrointestinal cancers that continue to be a leading cause of death worldwide. The cell line collection included esophageal carcinomas KYSE-30 and FLO-1, and colon cancer lines HCT-116 and RKO. For primary screening, the IC_50_ concentrations for κ- and λ-carrageenans had been identified with an MTT assay. The κo- and λo-carrageenans demonstrated a high range of cytotoxic activities for gastrointestinal cancer cell lines from 184 to 405 µg/mL, while equal cytotoxicity for RPE-1 cells requires much higher concentrations, 728 and 615 µg/mL for κo- and λo-carrageenans, respectively (Figure 3, Table S1). This fact led us to the idea that κo- and λo-carrageenans could have selective antitumor activities, which we tried to verify in the following experiments.

### 2.3. Effect of κo- and λo-Carrageenans on the Cell Cycle

To rule out the possibility of non-specific effect on normal cells, the cytotoxic, cell cycle and apoptosis assays were performed with the human non-transformed retinal pigmented epithelial RPE-1 cells immortalized by TERT1 transgene. First, we determined the IC_50_ concentration of κo- and λo- for KYSE-30, FLO-1, HCT-116, RKO, and RPE-1 cells (Figure 3). The major regulatory events leading to the reduction of proliferation occur during the cell cycle. To observe the effects of κo- and λo-carrageenans on the cell cycle we performed a flow cytometry analysis with Hoechst 33342. The cancer cell lines KYSE-30, FLO-1, HCT-116, RKO, showed delays in cell cycle progression upon treatment with κo- and λo-carrageenans; however, RPE-1 cells were not affected by either carrageenan. 

**Figure 4:**
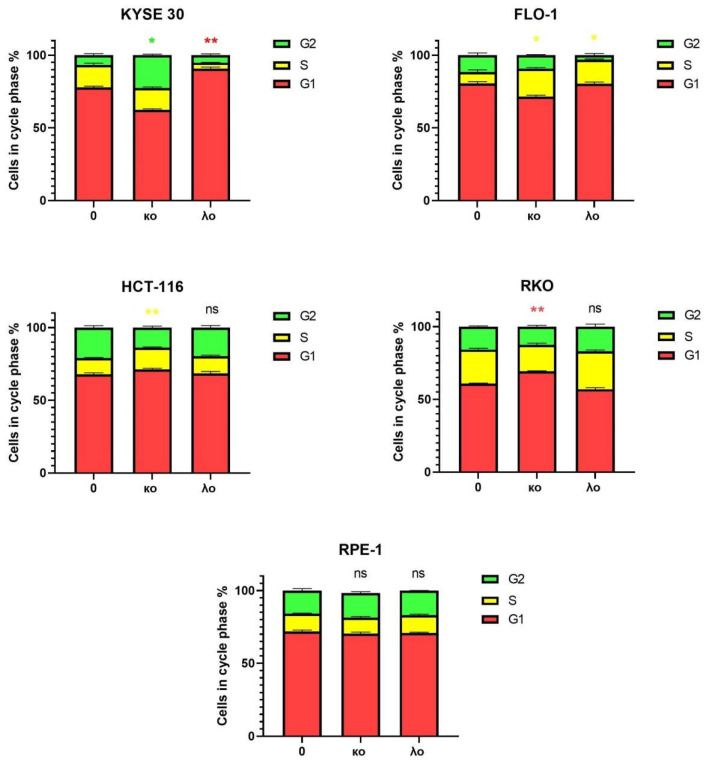
Cell cycle distribution of gastrointestinal cancer cell lines after treatment with κo-, λo- carrageenans. The results are expressed as % ratio of each phase. The color of * shows the difference in the color phase of the cell cycle. * < 0.05, ** < 0.01, ns—no significance.

We observed an accumulation of S phases in FLO-1 and HCT-116 cell lines after treatment with κo-carrageenan, while KYSE-30 demonstrated distinct G_2_/M delay. However, κo-carrageenan inhibited cell cycle in G_1_ stage in RKO cells (Figure 4, Table S2). Treatment with λo-carrageenan demonstrated S phase accumulation of FLO-1 cells, while KYSE-30 cells accumulated in G_1_ stage (Figure 4, Table S2). 

Thus, the results showed that low molecular weight carrageenans affect the cell cycle of cancer cells by inhibiting various phases. In particular, the κo-carrageenan mainly delayed cell cycle in the G_2_/M phase in KYSE-30, S phase in FLO-1 and HCT-116 cell lines and in G_1_ phase in RKO. The λo-carrageenan inhibited cell cycle in the S phase of FLO-1 and in the G_1_ phase of KYSE-30 esophageal cell lines (Figure 4, Table S2). 

An additional experiment was performed to observe S-G_2_-M cell accumulation in order to prove the effects of carrageenans on the cell cycle (Figure 4). In this experiment, the HCT-116 cells expressed a class of the GFP-fusion, i.e., a “destabilized GFP” (dGFP) (Figure S3). The approach that utilized destabilized GFP was described in [30]. This method is based on the expression of short FUCCI peptides (fluorescent ubiquitination-based cell-cycle indicator), GMNN and dGFP in a single ORF. The expression of these fusions may be visualized at the different stages of the cell cycle. HCT-116 cell line was transfected with plasmid encoding GFP fused with a 1–110 amino acid domain of DNA replication inhibitor (GMNN). FUCCI domains led to proteasomal degradation of GFP proteins associated with their C-terminal sequences, and the time of active functioning is strictly related to the duration of different cell cycle phases. GMNN is expressed during the S, G_2_, and M periods, and the GFP-GMNN fusion causes the cells to be green in the S-G_2_-M phases of the cell cycle. Thus, the cells stopping in S, G_2_, or M periods showed an accumulation of fluorescent signals (Figure S3). In this experiment, we observed an accumulation of GMNN-GFP-positive cells upon treatment with κo-carrageenan (Figure S3).

The average percentage of GFP/DAPI HCT-116-GMNN-GFP cells treated with κo-carrageenans was 17.4 ± 3.1. Statistical analysis was performed using one-way ANOVA. 

Summarized data correlate with cell cycle analysis and support our observation that κo-carrageenan treatment stimulates the S/G_2_/M cell cycle inhibition.

### 2.4. κo- and λo-Carrageenans Induce Apoptosis on Human Colon Cancer and Esophageal Carcinoma Cell Lines

**Figure 5:**
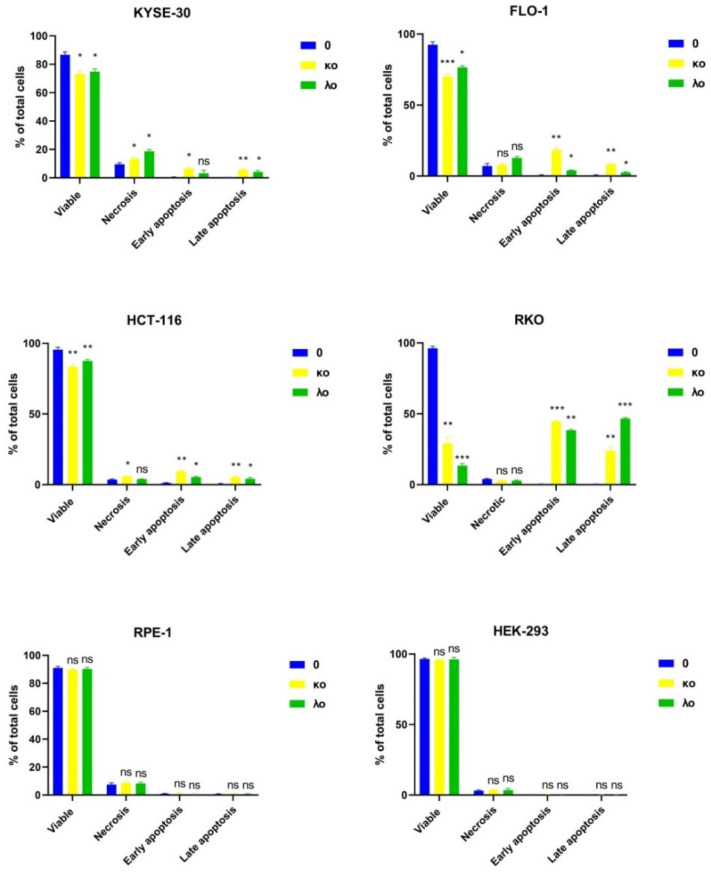
Apoptosis level in cancer cell lines KYSE-30, FLO-1, HCT-116, RKO and RPE-1 after treatment with κo- and λo-carrageenans for 48 h at IC_50_ concentrations. The results are expressed as columns and number of cells in % as groups of cells: viable, necrosis, early apoptosis, late apoptosis. * < 0.05, ** < 0.01, *** < 0.001, ns—no significance.

We observed induction of apoptosis after κo- and λo-carrageenans treatment on human colon cancer and esophageal carcinoma cell lines. The colon carcinoma cell line RKO revealed late apoptosis upon λo-carrageenan treatment, while after treatment with the κo-carrageenan accumulation of early apoptotic cells was observed instead. The early apoptotic cells were also detected in the HCT-116 colorectal carcinoma upon treatment with λo-carrageenan; however, treatment with κo-carrageenans induced late apoptosis and early apoptosis. In the esophagus cancer lines KYSE-30 and FLO-1, when exposed to κo- and λo- carrageenan, 10% of cells exhibited necrosis. Furthermore, upon treatment with κo-carrageenan, a large percentage of cells in the stage of early apoptosis were revealed (Figure 5).

The FLO-1 esophageal adenocarcinoma cell line showed an accumulation of necrotic cells upon treatment with λo-carrageenan. Cells treated with κo-carrageenan were at the stage of early apoptosis. Both κo- and λo-carrageenans did not have an effect on RPE-1 at all studied concentrations (Figure 5).The average percentage of early and late apoptotic KYSE-30 cells treated with κo-carrageenan was a 19.5 and 24.9 fold increase compared to negative control, subsequently, while KYSE-30 cells demonstrated high fold change with only λo-carrageenan. Early apoptotic FLO-1 cells influenced with κo-carrageenan have showed a similar picture; for λo-carrageenan treated cells both carrageenans led to the accumulation of early and late apoptosis. We observed similar effects on HCT-116 cells. As for the RKO cell line, the average percentage of early and late apoptotic cells after incubation with carrageenans increased dramatically. No significant changes were found in RPE-1 and HEK-293 cells incubated with both κo- and λo-carrageenans (Figure 5, Table S3, Figure S4).

To summarize, both κo- and λo-carrageenans have been proven to induce apoptosis in different cell lines. However, the high apoptosis induction is evident only in adenocarcinoma RKO cells upon treatment with κo- and λo-carrageenans. At the same time, in the FLO-1 cell line, only κo-carrageenan showed an apoptosis-inducing ability. 

### 2.5. Gastrointestinal Cancer Cells Treated with κo- and λo-Carrageenans Demonstrated a Reduction of CDK2 and E2F2

**Figure 6:**
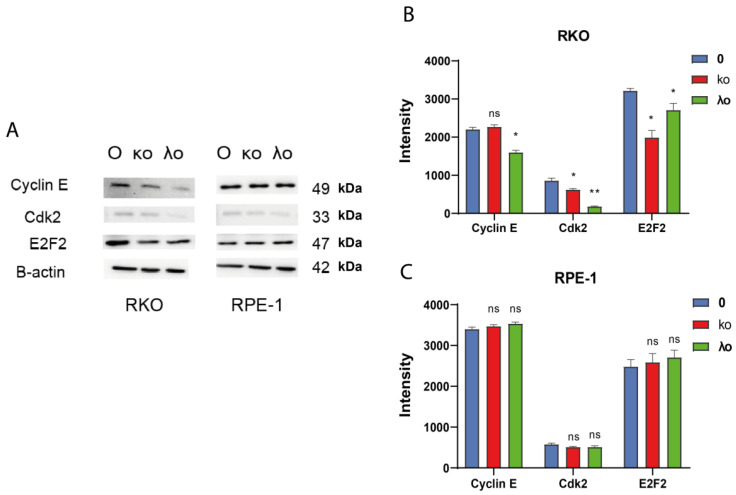
Western blot analysis of Cyclin E, Cdk2, E2F2 expression in RKO cells treated with κo- and λo-carrageenans. (**A**) Expression of cell cycle proteins Cyclin E, Cdk2, E2F2 and β-actin after treatment with κo-, λo-carrageenans for 48 h at IC_50_ concentrations in RKO and RPE-1 cell lines. (**B**) Statistical analysis of bands intensity of cyclin E, Cdk2, E2F2 proteins in the RKO colon adenocarcinoma cell line. (**C**) Statistical analysis of bands intensity of the proteins Cyclin E, Cdk2, E2F2 in RPE-1 * < 0.05, ** < 0.01, ns—no significance.

To prove the cell cycle phenotype observed after treatment with carrageenans we performed a western blot analysis with common cell cycle markers Cyclin E, CDK2, and E2F2 (B-actin was used as the reference protein). Analysis revealed that there was a significant decrease in Cyclin E, CDK2, and E2F2 proteins after treatment with κo- and λo- carrageenans (Figure 6, Table S4). However, on the RPE-1 cell line we did not observe significant changes.

λo-carrageenan inhibited all observed cell cycle proteins Cyclin E, Cdk2, and E2F2, while κo-carrageenan inhibits only Cdk2 and E2F2 proteins. However, no significant changes were found in RPE-1 cells treated with both κo- and λo- carrageenans (Table S4, Figure 6).

Thus, it was proven that κo- and λo-carrageenans induce cell cycle delay in RKO. After λo-carrageenan treatment, we observed the inhibition of Cyclin E, CDK2, and E2F2, while κo-carrageenan had an influence on the decrease of CDK2 and E2F2 proteins. We may guess that λo-carrageenan has a higher inhibitory potential effect on cell cycle than κo-carrageenan.

## 3. Discussion

The structure of κ-carrageenan is based on a disaccharide unit consisting of residues of 3-linked β-D-galactose, which has a sulfate group at C-4, and 4-linked 3,6-anhydro-D-galactose. λ-carrageenan is distinguished from other types of carrageenans by its high sulfation profile and the absence of 3,6-anhydrogalactose [26].

Initially obtained carrageenans are always polysaccharides, but by using ultrasound or chemical degradation they can be transformed into low molecular weight oligocarrageenans [15]. Tecson et al. showed that FT-IR analysis of ultrasound-degraded κ-carrageenan revealed a spectrum similar to the native κ-carrageenan polysaccharide. The study showed that its molecular weight is low due to a break along the chain along the O–glycosidic bond, which is prone to hydrolytic cleavage [31]. Our analysis of the IR spectra after ultrasound treatment revealed the preservation of functional groups and the molecular framework of native polysaccharides. This indicates that the major repeat units are retained.

Commonly used NMR analysis after the ultrasonic depolymerization of carrageenan can show structural changes [31]. ^13^C NMR spectra of low molecular weight sonicated κ-carrageenan showed common peaks with untreated κ-carrageenan, indicating that most of the native structure of κ-carrageenan was retained in the sonicated sample. It has been shown that, during depolymerization under ultrasound treatment, glycosidic bonds can break in random places, while the intensity of the change in intrinsic viscosity and molecular weight mainly depends on polymers concentration [31]. 

Some studies predict that the antiproliferative activity of oligocarrageenans increases with a decrease in their molecular weight [17,24]. Cytotoxic effect of high-molecular carrageenans was demonstrated on cancer lines such as HOS, Caco – 2, HepG2 and LM2 with IC_50_ greater than 1000 µg/mL [19,31,32,33], on other hand oligocarageenans exhibited the same effect at much lower IC_50_ ranging from 200 to 640 μg/mL. In the study by Ariffin et al., ultrasound degradation of λ-carrageenan and comprising different molecular weight carrageenans on cancer cell proliferation rate showed the growth of the antitumor activity with decreasing molecular size the effect of λ-carrageenan and it’s comprising different molecular weight carageenans represented inverse proportion between proliferation rates and their molecular size. Therefore, low molecular weight carrageenans convey an impression of more promising anticancer agents than high molecular weight [23,24,34]. This is in agreement with results observed in our study, that low molecular weight carrageenans of 0.2–22.5 kDa for κo- and 0.6–69.4 kDa for λo-carrageenan had a significant cytotoxic effect. In line with our results, Yuan, H. et al. observed the antiproliferative activity of native *Kappaphycus striatum* carrageenans. In this study, the κo-carrageenan with 1.2 kDa molecular weight showed higher antiproliferative activity than a 37.7 kDa κ-carrageenan. The study also showed that a wide range of concentrations—125, 250, and 500 μg/mL of κo- carrageenan—inhibits human nasopharyngeal carcinoma (KB), gastric carcinoma (BGC), and cervical cancer (HeLa) cell proliferation by 6.3 times, while κ-carrageenan inhibits cell proliferation by 2.7 times [17].

However, molecular weight is not the only feature that plays a role in the potential cytotoxicity of carrageenans. There are experimental examples where carrageenans caused cell cycle arrest [19,35]. In the study by Prasedya et al., the effects of carrageenans on the tumor cell cycle were observed using human cervical carcinoma cells (HeLa) and human umbilical vein endothelial cells (HUVEC) [35]. It was found that λ-carrageenan delays the cell cycle in the G_2_/M phase, while κ-carrageenan stops the cell cycle in both the G_1_ and G_2_/M phases. λ-carrageenan also suppressed the cell’s ability to divide, demonstrating a potential antiproliferative effect. HUVEC cells were not significantly affected by carrageenan. However, it was shown that κ-carrageenan in combination with selenium, known as κ-selenocarrageenan, blocked the human hepatoma cell cycle in the S-phase [36]. Degraded ι-carrageenan suppressed the Wnt/β-catenin signaling pathway and suppressed tumor growth. In addition, ι-carrageenan induced apoptosis and blocked the G_1_ phase in human osteosarcoma cells [33].

Morphological features of apoptosis were recorded in Caco-2 and HepG2 tumor cells of the human intestine after incubation with degraded κ-carrageenans. It is assumed that the toxicity of degraded carrageenans, realized through the mechanisms of apoptosis and inflammation, is associated with the production of reactive oxygen species (ROS) [37]. Signs of apoptosis were observed in A549 cancer cells upon treatment with a sulfated polysaccharide (420 kDa) isolated from *A. spicifera* [38]. κ-carrabiose, which has the highest cytotoxicity against several cancer lines, caused cell death by inducing arrest of the G_2_/M phase and apoptosis [16]. In another study, it was shown that the λ-carrageenan signaling pathway inhibited the proliferation of MDA-MB-231 cells by activating the proapoptotic genes of caspase-8, caspase-9, and caspase-3. This resulted in an increase in the level of active caspase-3 protein. In addition, carrageenan has the ability to disrupt mitochondrial function by altering the bax/bcl-2 expression ratio, which is considered an important element in the induction of apoptosis [39].

Cell cycle delays caused by disorders in levels of cyclin E, Cdk2 and EF2F expression are often associated with the progression of various human cancers [40]. E2F normally regulates the transition from the G_1_ phase to the S phase in the cell cycle. However, genome instability in cancer cells ultimately leads to a dysregulation of E2F2-dependent transcription. It has been shown that unregulated expression of E2F2 induces abnormal entry into S phase and apoptosis [41]. In our study, the isolated κo-carrageenan could inhibit cell cycle proteins, such as CDK2 and E2F2, consequently inducing cell cycle delay. κo-carrageenan delayed cell cycle in the G_2_ phase in KYSE-30, S phase in FLO-1 and HCT-116 cell lines, and inhibited G_1_ phase in RKO. λo-carrageenan delayed cell cycle in the S phase of FLO-1 and G_1_ in KYSE-30 cell lines. We observed strong induction of late apoptosis in the RKO cell line by λo-carrageenan, while κo-carrageenan showed a more pronounced early apoptosis induction. Moreover, the λo-carrageenan inhibited all observed cell cycle proteins Cyclin E, Cdk2 and E2F2, while the κo-carrageenan inhibits only Cdk2 and E2F2 proteins. Of course, these data cannot describe the detailed mechanism by which carrageenans inhibited the cell cycle differently in RKO and other cell types, specifically the lack of cell cycle inhibition after treatment with λo-carrageenan in RKO cells. However, observing apoptotic induction may lead us to a possible explanation for this discrepancy. A large proportion of cells exposed to λo-carrageenan go into apoptosis immediately, by passing cell cycle arrest, so such dramatic induction of apoptosis in RKO cells does not allow us to see statistically significant changes in the cell cycle in the presented experiment. In our future study we plan to determine targets of λo- and κo-carrageenans in the cell cycle regulation pathways and finally explain the detailed mechanisms of cell cycle delay caused by λo- and κo-carrageenans.

Today, cell cycle regulators are considered to be attractive targets in cancer therapy. In this work, it was shown that λo-carrageenan is able to reduce the expression of Cyclin E. There was also a significant decrease in the expression of cyclin-dependent kinase-2 after treatment with κo- and λo-carrageenans. At the same time, the decrease in expression after the λo-carrageenan was more pronounced than after the oligosaccharide κ-carrageenan. As for the E2F2 protein, a significant decrease in expression was observed after treatment with κo- and λo-carrageenans.

In summary, the data presented in this study shows for the first time the cell cycle delay upon treatment with carrageenans and further experiments will focus on mechanisms that underline these phenotypical effects. We also suggest carrageenans from *Chondrus armatus* could be a potential source for tumor suppressive molecules with cytostatic properties.

## 4. Materials and Methods

### 4.1. Obtaining Polysaccharides and Their Low Molecular Weight Derivatives

Red algae *Chondrus armatus* were collected at the Peter the Great Bay in the Sea of Japan. Samples of κ- and λ-carrageenan oligosaccharides isolated from the polysaccharides of the red seaweed *Chondrus armatus* by ultrasonic depolymerization were used in this project. 

Briefly, the dried algae were soaked in 96% ethanol and then subjected to triple aqueous extraction. The obtained extracts were combined and filtered, centrifuged at 2500× *g*, 10 °C for 20 min, and concentrated. Polysaccharides were precipitated with a threefold volume of 95% ethanol; the precipitate was filtered off and lyophilized. The resulting substance was a fraction of the total carrageenans. A 4% KCl solution (Helicon, Russia) was added to the polysaccharide solution, followed by centrifugation (10,000× *g*, 30 min, 4 °C). The obtained supernatant—KCl-soluble fraction—was subjected to ultrafiltration through a transverse Vivaflow 200 cassette (Sartorius, Göttingen, Germany); the resulting solution was concentrated under vacuum and freeze-dried. The resulting fraction was λ-carrageenan (λ-polysaccharide). The gel—KCl-insoluble fraction—was dialyzed for 5 days against 0.9% NaCl solution, then filtered through a Vivaflow 200 transverse cassette, and the solution was concentrated and lyophilized. The resulting fraction was κ- carrageenan (κ-polysaccharide).

For depolymerization of initial κ- and λ-carrageenan polysaccharides, sonication was performed using a Sonopuls HD 2200 homogenizer (Bandelin, Berlin, Germany). The duration of sonication was 1.5 h at amplitude of 40%, and the operation intervals were 8 s; sound frequency—20 kHz. The resulting samples of oligosaccharides (κo- and λo-) carrageenans were dialyzed and freeze-dried (Figure 2a).

### 4.2. Structural Features and Molecular Weight

The chemical structure of carrageenans isolated from the red alga *Chondrus armatus*, as well as their low molecular weight derivatives, was confirmed by Infrared spectroscopy. IR spectra were recorded on an IR Affinity-1S spectrometer (Shimadzu, Japan) using an attenuated total internal reflection attachment. The determination was carried out under the following conditions: spectral range in the range of 2000–600 cm^−1^, resolution 4 cm^−1^, number of scans 64. The LabSolutions IR 2.13 software (Shimadzu, Tokyo, Japan) was used to analyze the spectra (Figure 1B).

An NMR spectrum was obtained using an Avance II 400 NMR spectrometer (Bruker, Berlin, Germany) resonating at 100 MHz at 70 °C. The concentration of the samples was 5–7 mg of polysaccharide/mL of D_2_O. The 13C NMR analysis was performed in 36,000 scans.

The molecular weight distribution was determined by high performance size exclusion chromatography on a Shimadzu LC-20AD chromatograph equipped with a Shodex OHpak SB-804MHQ analytical column (Shimadzu, Kyoto, Japan). A 0.1 M NaNO_3_ solution was used as the mobile phase with a flow rate of 0.8 mL/min. To determine the molecular weights of the analyzed samples of carrageenans, they were built according to standard samples of pullulans (Figure 2b).

### 4.3. Cell Cultures

Cancer cell lines KYSE-30 (squamous cell carcinoma), FLO-1 (esophageal adenocarcinoma), HCT-116 (colorectal carcinoma), RKO (colon carcinoma), HEK-293 (kidney of a human embryo), and RPE-1 (retinal pigment epithelial cells) were selected for the experiments. All cell lines were cultured in DMEM + GlutaMAX media (Gibco, Waltham, MA, USA) containing 10% fetal bovine serum (Gibco, USA), gentamicin (100 μg/mL) (Dalkhimpharm, Khabarovsk, Russia), 1% sodium pyruvate (Gibco, USA), and amphotericin B (250 μg/mL) (Gibco, USA), in a CO_2_ incubator at 37 °C in the humid atmosphere 5% CO_2_.

### 4.4. IC_50_ Concentration

IC_50_ is the concentration of inhibitors at which 50% inhibition of its activity is achieved. Cells for IC_50_ determination were inoculated in a 96-well plate in the amount of 1 × 10^4^ cells and cultured overnight at 37 °C in CO_2_ atmosphere (5%). After incubation, κo- and λo- carrageenans were added to the plate at various concentrations (100, 200, 300, 400, 500, 600, 700, 800, 900, 1000 μg/mL), and then the cells were cultured under the same conditions for 48 h, respectively. The result was determined using the 3-(4,5-dimethylthiazol-2-yl)-2,5-diphenyltetrazolium bromide (MTT) assay and multifunctional plate reader Cytation 5 (BioTek, Broadview, IL, USA); the absorption was determined relative to the background absorption at 570 and 630 nm. The IC_50_ values were calculated using GraphPad Prism 8.1 software. We considered substances with IC_50_ values in the range of 100–1000 µg/mL to be moderately cytotoxic, while compounds with IC_50_ values exceeding 1000 µg/mL are considered non-toxic to cells [42].

### 4.5. Cell Cycle Assay

Cells were seeded into 12-well plates in an amount of 1 × 10^5^ cells and cultured in DMEM at 37 °C, 5% CO_2_ for 24 h. After incubation, the cells were treated with oligosaccharides of κ- and λ-carrageenans in IC_50_ concentrations determined previously and incubated for 48 h. After incubation, the cells were washed with PBS and stained with Hoechst 33342 (Thermo Fisher, Waltham, MA, USA) at a concentration of 10 μg/ml for 1 h. Cell media (DMEM) was used as a negative control.

The stained cells were analyzed for cell cycle distribution by a MoFlo Astrios EQ cell sorter (Beckman Coulter, Brea, CA, USA). Up to 10,000 events were accumulated. The forward (FSC) and lateral (SSC) scattering indices were adjusted, and singlets were separated on the FSC-H/FSC-A dot-plot. Hoechst-positive cells were separated on the DAPI-W/DAPI-logA dot-plot and later highlighted on a DAPI-logA histogram with cell cycle phases indication. Data collection and computer processing were performed using the Caluza software 2.1 (Beckman Coulter, USA).

### 4.6. Detection of S-G2-M Phase with Cell-Cycle Censor GEMNN_GFP

HCT-116 cells were seeded in 24-well plates with a density of 1 × 10^5^ cells/well and incubated for 24 h before transfection. For transient transfection, the 5 micrograms of pCX_GEMN_GFP plasmid (Supplementary Materials) and 15 micrograms of PEI were incubated with cells for 4 h in 500 µL serum-free media, the serum-free media were replaced by fresh media containing 10% FBS, and the cells were cultured for another 24 h before fluorescence microscope imaging for transfection efficiency. The rate of GFP-positive cells was measured using Synergy™ HTX Multi-Mode Microplate Reader (BioTek, USA). The number of GFP-positive and GFP-negative cells was counted in each field with a threshold of 7000. The cell size was restricted from 5 μm up to 100 μm. Samples were acquired in at least 9 separate fields.

### 4.7. Apoptosis Assay

Cells were seeded into 12-well plates in an amount of 1 × 10^5^ cells and cultured in DMEM at 37 °C, 5% CO_2_ for 24 h. For the next day, the cells were treated with oligosaccharides of κ- and λ-carrageenans in IC_50_ concentrations determined previously, and incubated for 48 h. After incubation, the cells were washed with PBS and 100 μL of binding buffer was added (10 mm HEPES/NaOH, pH 7.4, 140 mm NaCl and 2.5 mm CaCl_2_). Then, FITC-annexin V (10 μL) was added to the cells, followed by the addition of 10 μL PI (50 μg/mL PBS) from the ApoDETECT Annexin V-FITC Kit (Thermo Fisher, USA), incubated with Annexin V-FITC at 25 °C for 5 min, then washed again with PBS and incubated with PI for 10 min in the dark at 4 °C and then analyzed by flow cytometry.

The stained cells were analyzed for apoptosis by a MoFlo Astrios EQ cell sorter. Up to 10,000 events were accumulated. The detectors of forward (FSC), side (SSC) scattering Annexin V-FITC (488–513/26 nm), and PI (561–614/20) were adjusted. The following controls were used to set the quadrant boundaries: unstained cells, cells stained with Annexin V-FITC only (no PI), and cells stained with PI only (no Annexin V-FITC). The number of living cells was determined in the lower left square (Annexin-/PI-), apoptotic (Annexin +/PI−) cells were determined in the lower right square, and cells in late apoptosis (Annexin +/PI +) were estimated in the upper right square of the histogram. Data collection and computer processing were performed using the Kaluza software (Beckman Coulter, USA).

### 4.8. Western Blot

Western blot assay was used to determine the changes in expressions of cell cycle proteins after treatment with carrageenans. The cells were inoculated onto 6-well plates and treated with IC_50_ concentrations of κo- and λo-carrageenans for 48 h, and the whole protein was obtained using a cold RIPA lysis buffer. The protein concentration in the samples was determined by the Bradford method. BSA (0.125 mg/mL; 0.250 mg/mL; 0.500 mg/mL; 0.750 mg/mL, 1.0 mg/mL) was used as a standard.

The samples were subjected to 7% SDS-PAGE and transferred to PVDF membranes in a Tris-glycine buffer at 150 mA. After protein transfer, the membranes were blocked in 5% skimmed milk for 1 h at room temperature, then washed three times with PBST. Immunohybridization was performed using primary antibodies cyclin E, Cdk2, E2F2 (Cloud-Clone Corp., Wuhan, China) and b-actin (Abcam, Cambridge, MA, USA). The membrane was incubated in 10 mL of primary antibodies (diluted 1:1000) at room temperature for 2 h. Then, the PVDF membrane was washed with PBST three times and treated with secondary antibodies (at a dilution of 1:10,000) at room temperature for 1 h. The proteins were visualized by a BIORAD gel-documenting system (Hercules, CA, USA) and quantified by ImageLab program (Cleveland, OH, USA) by comparing it to the corresponding band intensity of β-actin.

### 4.9. Statistical Analysis

All data are presented as mean ± SD from a minimum of three independent experiments. Comparisons for each experiment were performed using one-way ANOVA in GraphPad Prism 6.0 (GraphPad Software, USA). A P-value less than 0.05 was considered statistically significant.

## 5. Conclusions

Here, we have isolated, characterized and reported on antitumor in vitro activity for κo- and λo-carrageenans from marine alga *Chondrus armatus*. The isolated κo-carrageenan could inhibit cell cycle proteins, such as CDK2 and E2F2, consequently inducing cell cycle delay. κo-carrageenan blocked cell cycle in the G_2_ phase in KYSE-30, S phase in FLO-1 and HCT-116 cell lines and inhibited G_1_ phase in RKO. λo-carrageenan inhibited cell cycle in the S phase of FLO-1 and G_1_ in KYSE-30 esophageal cell lines. Moreover, after λo-carrageenan treatment, we observed a significant decrease in Cyclin E, CDK2 and E2F2 proteins. Additionally, κo- and λo-carrageenans selectively induced apoptosis of human colon RKO.

## Figures and Tables

**Table 1:** ^13^C NMR Signals (Δ ppm) in the Spectrum of κ- and λo- carrageenans.

Type of Carrageenan	Monosaccharide Unit	^13^C Signals (ppm.)					
		C-1	C-2	C-3	C-4	C-5	C-6
κ [28]	G4S	102.5	69.6	78.9	74.1	74.8	61.3
κo [29]		103.1	70.09	79.04	74.2	75.5	61.9
κ [28]	DA	95.3	69.9	79.2	78.3	76.8	69.5
κo [29]		95.82	70.48	79.83	77.8	77.4	70.2
λ [28]	G2S	103.4	77.4	75.8	64.2	74.2	61.3
λo		101.1, 102.5, 103.1, 104.0	78.7, 76.4	76.3	64.7	74.4, 74.2	60.4, 60.6
λ [28]	D2S,6S	91.6	74.8	69.5	80.3	68.7	68.1
λo		91.6	75.1	69.6	80.4	68.9, 68.6	68.1

**Table 2:** Molecular weights of κ-, κo-, λ-, λo- carrageenans. Mn—number average molecular mass, Mw—weight average molecular mass, Mw/Mn—polydisperse index.

	Mn	Mw	Mw/Mn
κ-polysaccharide	678.8 kDa	783.9 kDa	3.09
κo-oligosaccharide	0.9 kDa	3.93 kDa	3.96
λ-polysaccharide	844.2 kDa	851.4 kDa	4.19
λo-oligosaccharide	21.4 kDa	69.4 kDa	3.24

## Data Availability

Not applicable.

## Supplementary Materials

The following are available online at https://www.mdpi.com/article/10.3390/md20120741/s1: Figure S1: ^13^C NMR spectrum of the fraction of isolated κo-carrageenan; Figure S2: ^13^C NMR spectrum of the fraction of isolated λo-carrageenan; Table S1: The IC_50_ concentrations of κo- and λo-carrageenans; Table S2: Cell cycle analysis of KYSE-30, FLO-1, HCT-116, RKO and RPE-1 cells after κo- and λo-treatment; Figure S3: Distribution of the cell cycle by GMNN_GFP fusions expression; Table S3: Apoptosis analysis of KYSE-30, FLO-1, HCT-116, RKO and RPE-1 cells after κo- and λo-treatment; Figure S4: Representative flow cytometry plots of Annexin-FitcV/PI apoptotic assay; Table S4: Statistical analysis of cyclin E, Cdk2, E2F2 proteins in the RKO colon adenocarcinoma cell line and in immortalized cells of the pigmented epithelium RPE-1 in comparison with vehicle (DMEM) treatment.
