# Supplementary material for: κ- and λ-Carrageenans from Marine Alga Chondrus armatus Exhibit Anticancer In Vitro Activity in Human Gastrointestinal Cancers Models
Source: Mar Drugs. 2022 Nov 25;20(12):741. doi: 10.3390/md20120741 (PMC9783017; doi:10.3390/md20120741)
Supplement: Supplementary file 1 [file marinedrugs-20-00741-s001.zip › marinedrugs-1940353-supplementary.pdf]

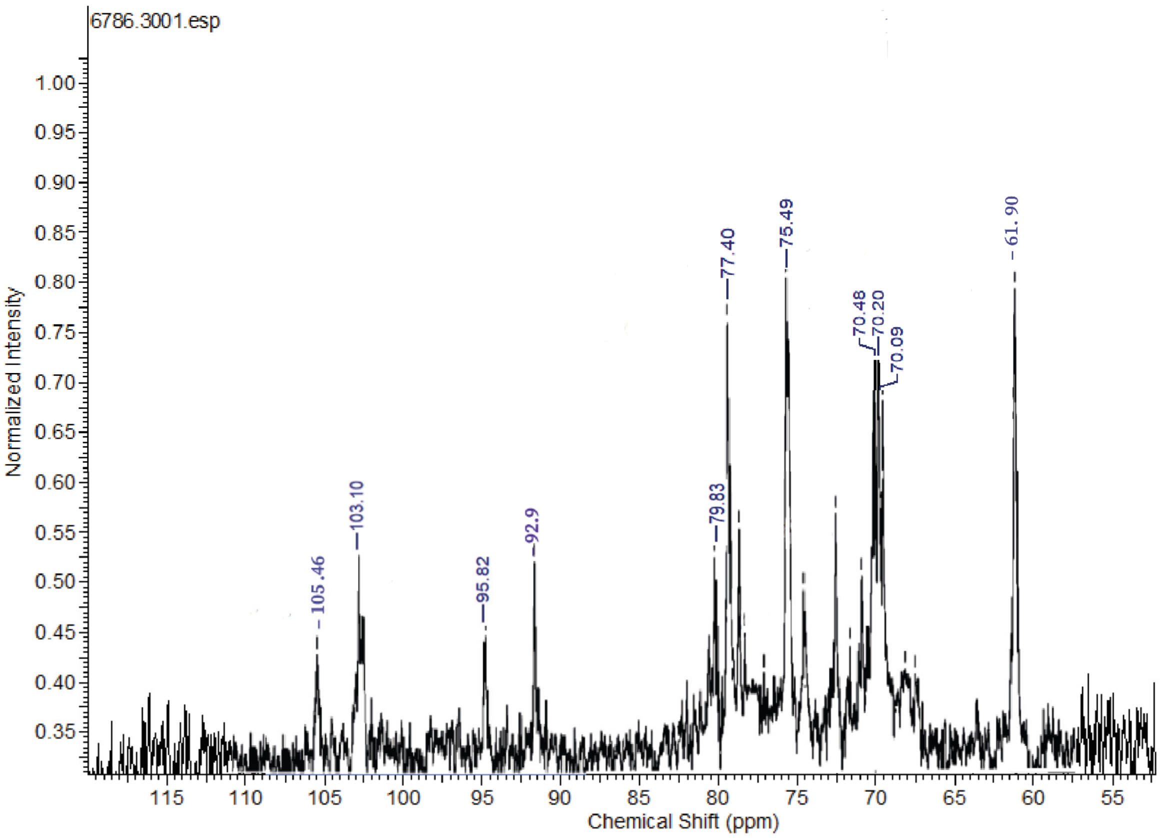

Supplementary Figure S1. ^{13}C NMR spectrum of the fraction of isolated κο-carrageenan [Kalitnik, 2013 [29].

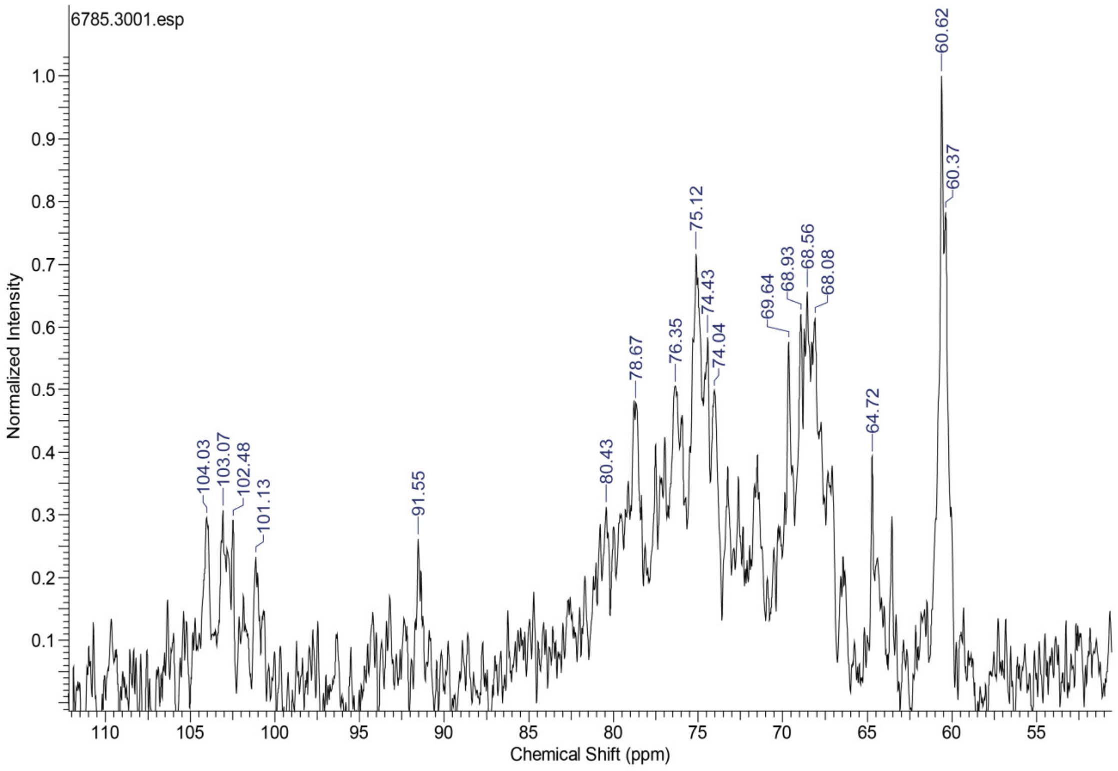

Supplementary Figure S2. ^{13}C NMR spectrum of the fraction of isolated λο-carrageenan.

Supplementary Table S1. The IC_{50} concentrations of κo- and λo- carrageenans.

**IC 50 µg/ml**	**KYSE-30**	**FLO-1**	**HCT-116**	**RKO**	**RPE-1**
**κo-**	394	405	347	350,6	728
**λo-**	352	184	206	248,3	615

Supplementary Table S2. Cell cycle analysis of KYSE-30, FLO-1, HCT-116, RKO and RPE-1 cells after κo-, λo- treatment. Average percentages, standard deviations (± SD), fold change in comparison the control and p values of 2-way Anova test with Sidak multiple comparisons (fold changes and p-values presented are calculated comparing the sample (κo-, λo-) to the untreated cells (0).

Exposure time, hrs	Cells	Phase	Sample	% of cells in cell cycle phase	(± SD)	Fold increase	p value
48	KYSE-30	G2/M	0	6.7	1.03	3.3	<0.005
			κo-	22.5	0.7		
		G1/S	0	77.8	0.82	1.16	<0.001
			λo-	90.76	1.01		
	FLO-1	S/G2	0	7.82	1.53		<0.01
			κo-	19.3	0.62	2.4	
			λo-	16.3	0.52	2.09	
	HCT-116	S/G2	0	11.2	0.44	1.32	<0.005
			κo-	14.9	0.51		
	RKO	G1/S	0	60.8	0.41	1.13	<0.005
			κo-	69.2	0.36		
	RPE-1	G2/M	0	15.87	1.34	ns	-
			κo-	16.82	0.88		
			λo-	16.87	0.08		

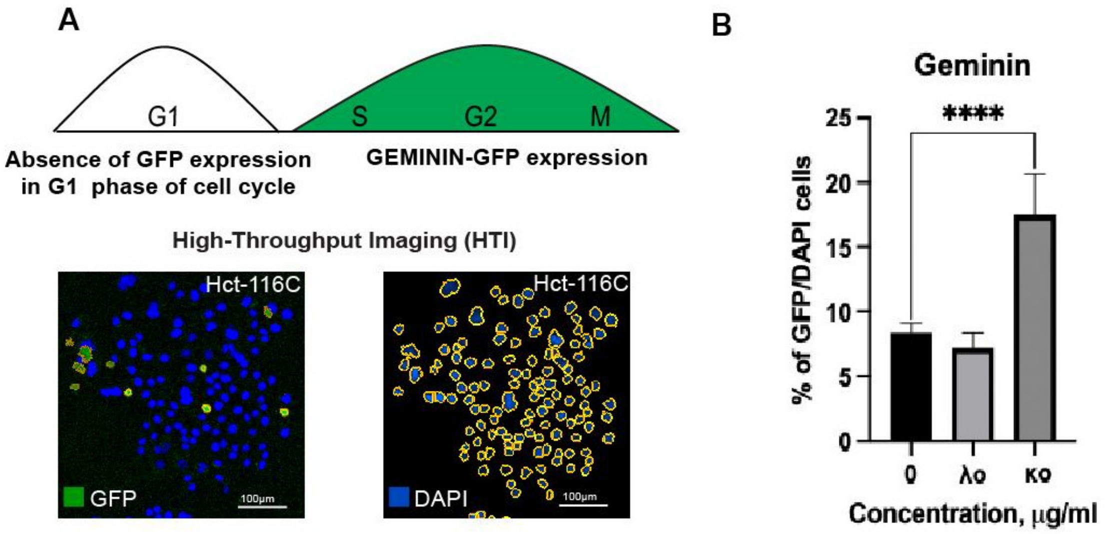

Supplementary Figure S3. Distribution of the cell cycle by GMNN_GFP fusions expression. (**a**) HCT-116 cells express GFP-fusions: GMININ-GFP is expressed in the S-G-M phases of the cell cycle. (**b**) Accumulation GMININ-GFP-positive cells upon treatment with κo- carrageenan.

Supplementary Table S3. Apoptosis analysis of KYSE-30, FLO-1, HCT-116, RKO and RPE-1 cells after κo-, λo- treatment. Average percentages, standard deviations (± SD), fold change in comparison the control and p values of 2-way Anova test with Sidak multiple comparisons (fold changes and p-values presented are calculated comparing the sample (κo-, λo-) to the untreated cells (0).

Cells	Stage	Treatment	% of cells in apoptotic stage	(± SD)	Fold increase	p value
KYSE-30	Early apoptosis	0	0.33	0.11	19.5	< 0,02
		κo-	6.45	1.11		
		λo-	3.19	2.17	ns	-
	Late apoptosis	0	0.22	0.04	24.9	<0,005
		κo-	5.49	0.47		
		λo-	4.16	0.91	18.9	<0,02
FLO-1	Early apoptosis	0	0.4	0.52	45.8	<0,005
		κo-	18.33	1.52		
		λo-	3.94	0.38	9.85	
	Late apoptosis	0	0.54	0.44	15.5	<0,05
		κo-	8.4	0.36		
		λo-	2.54	0.46	4.7	

HCT-116	Early apoptosis	0	1.23	0.25	7.58	<0,005
		κo-	9.33	0.57		
		λo-	5.26	0.64	4.27	<0,01
	Late apoptosis	0	0.51	0.47	10.25	<0,005
		κo-	5.23	0.68		
		λo-	4.00	1.00	7.84	<0,02
RKO	Early apoptosis	0	0.55	0.22	80.9	<0,002
		κo-	44.5	0.45		
		λo-	38.2	0.64	69.45	
	Late apoptosis	0	0.93	0.07	25.7	<0,0,005
		κo-	23.9	2.8		
		λo-	46.7	0.64	50,2	<0,001
RPE-1	Early apoptosis	0	0.88	0.22	ns	-
		κo-	0.79	0.86		
		λo-	0.34	0.16		
	Late apoptosis	0	0.7	0.3	ns	-
		κo-	0.8	0.08		
		λo-	0.72	0.13		
HEK-293	Early apoptosis	0	0.35	0.06	ns	-
		κo-	0.44	0.21		
		λo-	0.31	0.135		
	Late apoptosis	0	0.113	0.07	ns	-
		κo-	0.31	0.135		
		λo-	0.1	0.07		

The data set was obtained by the average of three independent experiments for the κo- and λo-compounds.

Supplementary Figure S4. Representative flow cytometry plots of Annexin-FitcV/PI apoptotic assay.

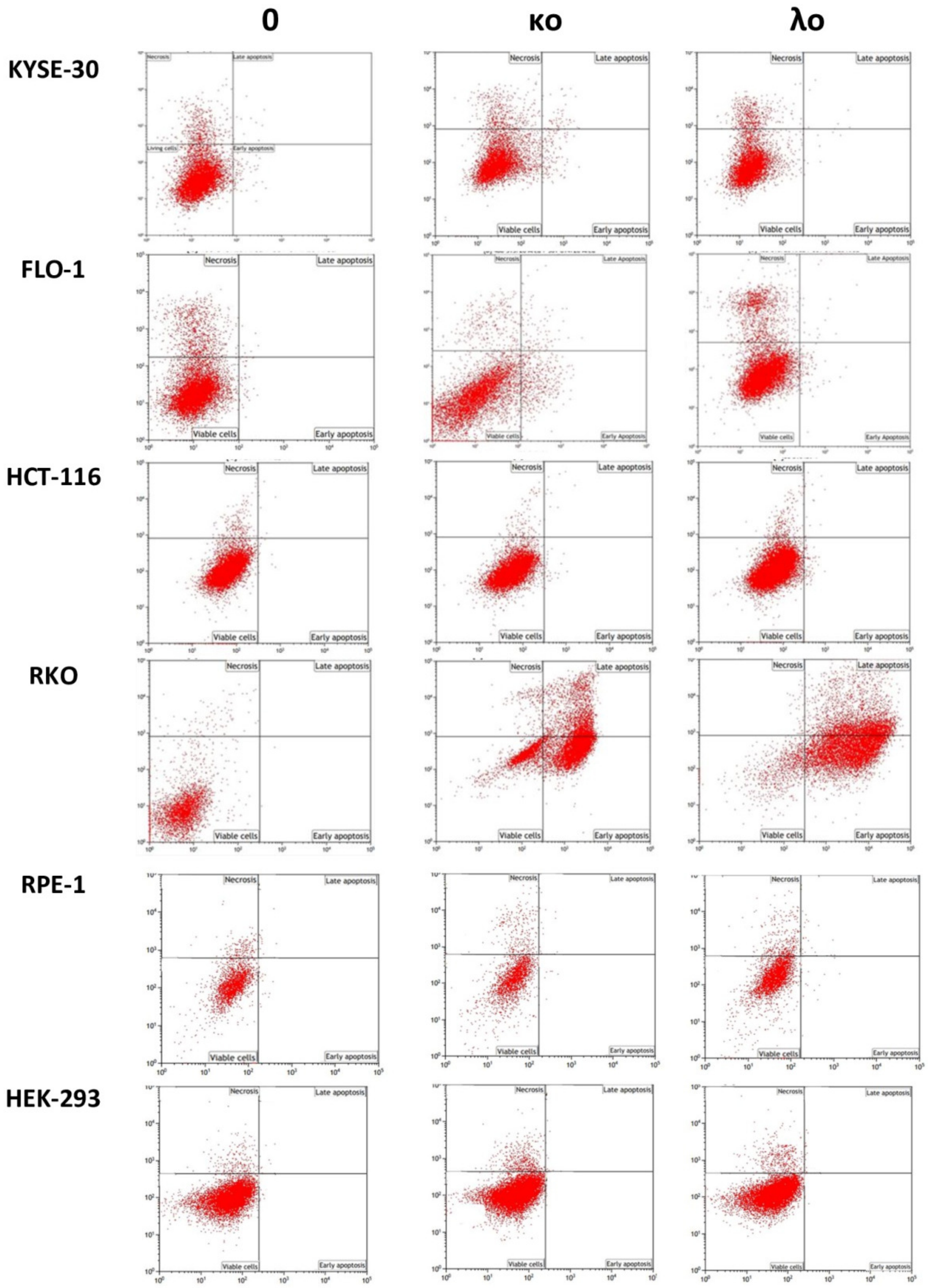

Supplementary Table S4. Statistical analysis of cyclin E, Cdk2, E2F2 proteins in the RKO colon adenocarcinoma cell line and in immortalized cells of the pigmented epithelium RPE-1 in comparison with vehicle (DMEM) treatment. Average percentages, standard deviations (± SD), fold change in comparison to the control and p values of 2-way Anova test with Sidak multiple comparisons (fold change and p-value presented are calculated comparing the sample (κo- and λo-) to the untreated cells (DMEM).

Cells	Protein	Treatment	quantitative density of protein	(± SD)	Fold decrease	p value
RKO	Cyclin E	0	2202	56.2	1.4	<0,0001
		λo-	1563	21.5		
	Cdk$_2$	0	857	66.3	1.37	<0,0001
		κo-	622	29.6		
		λo-	181	9.4	4.7	
	E$_2$F$_2$	0	3214	64.2	1.64	<0,0001
		κo-	1955	42.7		
		λo-	2554	51.4	1.25	
RPE-1	Cyclin E	0	3403	47,5	ns	-
		κo-	3472	38,7		
		λo-	3535	40,06		
	Cdk$_2$	0	857	66	ns	-
		κo-	622	29,5		
		λo-	181	9,4		
	E2F2	0	2480	172,7	ns	-
		κo-	2589	212		
		λo-	2707	177		

The data set was obtained by the average of three independent experiments for the κo- and λo-compounds.

The pCX_GEMN_GFP plasmid sequence:

GTCGACATTGATTATTGACTAGTTATTAATAGTAATCAATTACGGGGTCATTAGTTC
ATAGCCCATATATGGAGTTCCGCGTTACATAACTTACGGTAAATGGCCCGCCTGGCT
GACCGCCCAACGACCCCCGCCCATTGACGTCAATAATGACGTATGTTCCCATAGTAA
CGCCAATAGGGACTTTCCATTGACGTCAATGGGTGGACTATTTACGGTAAACTGCCC
ACTTGGCAGTACATCAAGTGTATCATATGCCAAGTACGCCCCCTATTGACGTCAATG
ACGGTAAATGGCCCGCCTGGCATTATGCCCAGTACATGACCTTATGGGACTTTCCTA
CTTGGCAGTACATCTACGTATTAGTCATCGCTATTACCATGGGTCGAGGTGAGCCCC
ACGTTCTGCTTCACTCTCCCCATCTCCCCCCCCTCCCCACCCCCAATTTTGTATTTATT
TATTTTTTAATTATTTTGTGCAGCGATGGGGGCGGGGGGGGGGGGGGCGCGCGCCA
GGCGGGGCGGGGCGGGGCGAGGGGCGGGGCGGGGCGAGGCGGAGAGGTGCGGCG
GCAGCCAATCAGAGCGGCGCGCTCCGAAAGTTTCCTTTTATGGCGAGGCGGCGGCG
GCGGCGGCCCTATAAAAAGCGAAGCGCGCGGCGGGCGGGAGTCGCTGCGTTGCCTT
CGCCCCGTGCCCCGCTCCGCGCCGCCTCGCGCCGCCCGCCCCGGCTCTGACTGACCG
CGTTACTCCCACAGGTGAGCGGGCGGGACGGCCCTTCTCCTCCGGGCTGTAATTAGC
GCTTGGTTTAATGACGGCTCGTTTCTTTTCTGTGGCTGCGTGAAAGCCTTAAAGGGCT
CCGGGAGGGCCCTTTGTGCGGGGGGGAGCGGCTCGGGGGGTGCGTGCGTGTGTGTG
TGCGTGGGGAGCGCCGCGTGCGGCCCGCGCTGCCCGGCGGCTGTGAGCGCTGCGGG
CGCGGCGCGGGGCTTTGTGCGCTCCGCGTGTGCGCGAGGGGAGCGCGGCCGGGGGC
GGTGCCCCGCGGTGCGGGGGGGCTGCGAGGGGAACAAAGGCTGCGTGCGGGGTGT
GTGCGTGGGGGGGTGAGCAGGGGGTGTGGGCGCGGCGGTCGGGCTGTAACCCCCCC
CTGCACCCCCCTCCCCGAGTTGCTGAGCACGGCCCGGCTTCGGGTGCGGGGCTCCGT
GCGGGGCGTGGCGCGGGGCTCGCCGTGCCGGGCGGGGGGTGGCGGCAGGTGGGGG
TGCCGGGCGGGGCGGGGCCGCCTCGGGCCGGGGAGGGCTCGGGGGAGGGGCGCGG
CGGCCCCGGAGCGCCGGCGGCTGTCGAGGCGCGGCGAGCCGCAGCCATTGCCTTTT
ATGGTAATCGTGCGAGAGGGCGCAGGGACTTCCTTTGTCCCAAATCTGGCGGAGCC
GAAATCTGGGAGGCGCCGCCGCACCCCCTCTAGCGGGCGCGGGCGAAGCGGTGCGG
CGCCGGCAGGAAGGAAATGGGCGGGGAGGGCCTTCGTGCGTCGCCGCGCCGCCGTC
CCCTTCTCCATCTCCAGCCTCGGGGCTGCCGCAGGGGGACGGCTGCCTTCGGGGGGG
ACGGGGCAGGGCGGGGTTCGGCTTCTGGCGTGTGACCGGCGGCTCTAGAGCCTCTG
CTAACCATGTTCATGCCTTCTTCTTTTTCCTACAGCTCCTGGGCAACGTGCTGGTTGT
TGTGCTGTCTCATCATTTTGGCAAAGAATTCGCCACCATGGTGAGCAAGGGCGAGGA
GCTGTTCACCGGGGTGGTGCCCATCCTGGTCGAGCTGGACGGCGACGTAAACGGCC
ACAAGTTCAGCGTGTCCGGCGAGGGCGAGGGCGATGCCACCTACGGCAAGCTGACC
CTGAAGTTCATCTGCACCACCGGCAAGCTGCCCGTGCCCTGGCCCACCCTCGTGACC

ACCCTGACCTACGGCGTGCAGTGCTTCAGCCGCTACCCCGACCACATGAAGCAGCA
CGACTTCTTCAAGTCCGCCATGCCCGAAGGCTACGTCCAGGAGCGCACCATCTTCTT
CAAGGACGACGGCAACTACAAGACCCGCGCCGAGGTGAAGTTCGAGGGCGACACC
CTGGTGAACCGCATCGAGCTGAAGGGCATCGACTTCAAGGAGGACGGCAACATCCT
GGGGCACAAGCTGGAGTACAACTACAACAGCCACAACGTCTATATCATGGCCGACA
AGCAGAAGAACGGCATCAAGGTGAACTTCAAGATCCGCCACAACATCGAGGACGGC
AGCGTGCAGCTCGCCGACCACTACCAGCAGAACACCCCCATCGGCGACGGCCCCGT
GCTGCTGCCCGACAACCACTACCTGAGCACCCAGTCCGCCCTGAGCAAAGACCCCA
ACGAGAAGCGCGATCACATGGTCCTGCTGGAGTTCGTGACCGCCGCCGGGATCACT
CTCGGCATGGACGAGCTGGATCCATCACACTGGCGGCCGCTCGAGATGAATCCCAG
TATGAAGCAGAAACAAGAAGAAATCAAAGAGAATATAAAGAATAGTTCTGTCCCAA
GAAGAACTCTGAAGATGATTCAGCCTTCTGCATCTGGATCTCTTGTTGGAAGAGAA
ATGAGCTGTCCGCAGGCTTGTCCAAAAGGAAACATCGGAATGACCACTTAACATCT
ACAACTTCCAGCCCTGGGGTTATTGTCCCAGAATCTAGTGAAAATAAAAATCTTGGA
GGAGTCACCCAGGAGTCATTTGATCTTATGATTAAAGAAAATCCATCCTCTCAGTAT
TGGAAGGAAGTGGCAGAAAAACGGAGAAAGGCGCTGTAAGAATTCACTCCTCAGGT
GCAGGCTGCCTATCAGAAGGTGGTGGCTGGTGTGGCCAATGCCCTGGCTCACAAAT
ACCACTGAGATCTTTTTCCCTCTGCCAAAAATTATGGGGACATCATGAAGCCCCTTG
AGCATCTGACTTCTGGCTAATAAAGGAAATTTATTTTCATTGCAATAGTGTGTTGGA
ATTTTTTGTGTCTCTCACTCGGAAGGACATATGGGAGGGCAAATCATTTAAAACATC
AGAATGAGTATTTGGTTTAGAGTTTGGCAACATATGCCATATGCTGGCTGCCATGAA
CAAAGGTGGCTATAAAGAGGTCATCAGTATATGAAACAGCCCCCTGCTGTCCATTCC
TTATTCCATAGAAAAGCCTTGACTTGAGGTTAGATTTTTTTTATATTTTGTTTTGTGTT
ATTTTTTTCTTTAACATCCCTAAAATTTTCCTTACATGTTTTACTAGCCAGATTTTTCC
TCCTCTCCTGACTACTCCCAGTCATAGCTGTCCCTCTTCTCTTATGAAGATCCCTCGA
CCTGCAGCCCAAGCTTGGCGTAATCATGGTCATAGCTGTTTCCTGTGTGAAATTGTT
ATCCGCTCACAATTCCACACAACATACGAGCCGGAAGCATAAAGTGTAAAGCCTGG
GGTGCCTAATGAGTGAGCTAACTCACATTAATTGCGTTGCGCTCACTGCCCGCTTTC
CAGTCGGGAAACCTGTCGTGCCAGCGGATCCGCATCTCAATTAGTCAGCAACCATA
GTCCCGCCCCTAACTCCGCCCATCCCGCCCCTAACTCCGCCCAGTTCCGCCCATTCTC
CGCCCCATGGCTGACTAATTTTTTTTATTTATGCAGAGGCCGAGGCCGCCTCGGCCT
CTGAGCTATTCCAGAAGTAGTGAGGAGGCTTTTTTGGAGGCCTAGGCTTTTGCAAAA
AGCTAACTTGTTTATTGCAGCTTATAATGGTTACAAATAAAGCAATAGCATCACAAA
TTTCACAAATAAAGCATTTTTTTCACTGCATTCTAGTTGTGGTTTGTCCAAACTCATC
AATGTATCTTATCATGTCTGGATCCGCTGCATTAATGAATCGGCCAACGCGCGGGGA

GAGGCGGTTTGCGTATTGGGCGCTCTTCCGCTTCCTCGCTCACTGACTCGCTGCGCTC
GGTCGTTCGGCTGCGGCGAGCGGTATCAGCTCACTCAAAGGCGGTAATACGGTTATC
CACAGAATCAGGGGATAACGCAGGAAAGAACATGTGAGCAAAAGGCCAGCAAAAG
GCCAGGAACCGTAAAAAGGCCGCGTTGCTGGCGTTTTTCCATAGGCTCCGCCCCCCT
GACGAGCATCACAAAAATCGACGCTCAAGTCAGAGGTGGCGAAACCCGACAGGACT
ATAAAGATACCAGGCGTTTCCCCCTGGAAGCTCCCTCGTGCGCTCTCCTGTTCCGAC
CCTGCCGCTTACCGGATACCTGTCCGCCTTTCTCCCTTCGGGAAGCGTGGCGCTTTCT
CAATGCTCACGCTGTAGGTATCTCAGTTCGGTGTAGGTCGTTCGCTCCAAGCTGGGC
TGTGTGCACGAACCCCCCGTTCAGCCCGACCGCTGCGCCTTATCCGGTAACTATCGT
CTTGAGTCCAACCCGGTAAGACACGACTTATCGCCACTGGCAGCAGCCACTGGTAA
CAGGATTAGCAGAGCGAGGTATGTAGGCGGTGCTACAGAGTTCTTGAAGTGGTGGC
CTAACTACGGCTACACTAGAAGGACAGTATTTGGTATCTGCGCTCTGCTGAAGCCAG
TTACCTTCGGAAAAAGAGTTGGTAGCTCTTGATCCGGCAAACAAACCACCGCTGGTA
GCGGTGGTTTTTTTGTTTGCAAGCAGCAGATTACGCGCAGAAAAAAAGGATCTCAA
GAAGATCCTTTGATCTTTTCTACGGGGTCTGACGCTCAGTGGAACGAAAACTCACGT
TAAGGGATTTTGGTCATGAGATTATCAAAAAGGATCTTCACCTAGATCCTTTTAAAT
TAAAAATGAAGTTTTAAATCAATCTAAAGTATATATGAGTAAACTTGGTCTGACAGT
TACCAATGCTTAATCAGTGAGGCACCTATCTCAGCGATCTGTCTATTTCGTTCATCCA
TAGTTGCCTGACTCCCCGTCGTGTAGATAACTACGATACGGGAGGGCTTACCATCTG
GCCCCAGTGCTGCAATGATACCGCGAGACCCACGCTCACCGGCTCCAGATTTATCAG
CAATAAACCAGCCAGCCGGAAGGGCCGAGCGCAGAAGTGGTCCTGCAACTTTATCC
GCCTCCATCCAGTCTATTAATTGTTGCCGGGAAGCTAGAGTAAGTAGTTCGCCAGTT
AATAGTTTGCGCAACGTTGTTGCCATTGCTACAGGCATCGTGGTGTCACGCTCGTCG
TTTGGTATGGCTTCATTCAGCTCCGGTTCCCAACGATCAAGGCGAGTTACATGATCC
CCCATGTTGTGCAAAAAAGCGGTTAGCTCCTTCGGTCCTCCGATCGTTGTCAGAAGT
AAGTTGGCCGCAGTGTTATCACTCATGGTTATGGCAGCACTGCATAATTCTCTTACT
GTCATGCCATCCGTAAGATGCTTTTCTGTGACTGGTGAGTACTCAACCAAGTCATTC
TGAGAATAGTGTATGCGGCGACCGAGTTGCTCTTGCCCGGCGTCAATACGGGATAAT
ACCGCGCCACATAGCAGAACTTTAAAAGTGCTCATCATTGGAAAACGTTCTTCGGGG
CGAAAACTCTCAAGGATCTTACCGCTGTTGAGATCCAGTTCGATGTAACCCACTCGT
GCACCCAACTGATCTTCAGCATCTTTTACTTTCACCAGCGTTTCTGGGTGAGCAAAA
ACAGGAAGGCAAAATGCCGCAAAAAAGGGAATAAGGGCGACACGGAAATGTTGAA
TACTCATACTCTTCCTTTTTCAATATTATTGAAGCATTTATCAGGGTTATTGTCTCAT
GAGCGGATACATATTTGAATGTATTTAGAAAAATAAACAAATAGGGGTTCCGCGCA
CATTTCCCCGAAAAGTGCCACCTG
